# How does agricultural intensification impact insect diversity and abundance in the palm groves of Algeria's Sahara?

**DOI:** 10.3897/BDJ.13.e170804

**Published:** 2025-12-04

**Authors:** Wahiba Boukhelouf, Imene Benzina, Abdelkrim Si Bachir, Faiza Marniche

**Affiliations:** 1 Department of Agronomy, Faculty of Sciences, University of Mohamed Khider, Biskra, Algeria Department of Agronomy, Faculty of Sciences, University of Mohamed Khider Biskra Algeria; 2 Laboratory Biodiversity, Biotechnology and Sustainable Development LBBDD, Faculty of Nature and Life Sciences, University Batna 2, Batna, Algeria Laboratory Biodiversity, Biotechnology and Sustainable Development LBBDD, Faculty of Nature and Life Sciences, University Batna 2 Batna Algeria; 3 National Veterinary School ENSV, Rabie Bouchama, El Alia, Algiers, Algeria National Veterinary School ENSV, Rabie Bouchama, El Alia Algiers Algeria

**Keywords:** agricultural intensification, palm grove, insects, diversity, Sahara

## Abstract

This study investigated the impact of agricultural intensification (AI) on insect diversity and abundance in date palm orchards (*Phoenix dactylifera* L.) in north-eastern Algeria under a Saharan climate. Insect sampling was conducted using various traps from September 2020 to August 2021 in three orchards along a gradient of agricultural intensification (low, medium and high). The study involved the examination of 5,633 insect specimens representing 267 species spanning 11 orders, 93 families and 195 genera. The results indicate that the diversity and abundance of insects are highest in moderately managed palm groves, followed by highly intensified groves and are lowest in non-intensified groves. The species composition is significantly more similar between moderately and highly intensified palm groves. The degree of agricultural intensification has a diferential affect on various insect groups, favouring the diversity or abundance of some, while limiting that of others. This is the case of Diptera and Hymenoptera, which are sensitive to agricultural intensification. Globally, moderate intensification has a more positive impact on the diversity and abundance of various insect orders. This highlights the need for the rational and controlled use of chemical inputs in order to preserve the diversity of insects in palm groves where they provide vital ecosystem services.

## Introduction

The intensification of agriculture is important to wrestle food insecurity, population growth and environmental degradation (Sariyev et al. 2025). This is pursued through two main strategies: expanding cultivable land and improving farming practices (Milleville and Serpantié 1994). Agricultural development programmes emphasising intensification were crucial during the green revolutions of the 1960s and remain vital for ongoing development in sub-Saharan Africa (Clay 2018). Since the 1960s, agricultural intensification (AI) has boosted crop yields, but negatively impacts pollinating insects and biodiversity, affecting both beneficial insects and pests (Deguines et al. 2014).

Up to 40% of insects are at risk of extinction due to habitat loss caused by agriculture and urbanisation, as well as pesticide pollution (Sánchez-Bayo and Wyckhuys 2019). Although AI leads to higher yields per labour unit (Serra 1986), the practices harm biodiversity by adversely affecting key predator populations (Jacquot 2012). Intensified rice production in Asia has resulted in the emergence of multiple pests (Wilby and Thomas 2002). Intensive management practices in orchards and meadows of the northern Italian Alps negatively affect soil invertebrate communities (Guariento et al. 2020). Similarly, in olive groves, increased agricultural management intensity leads to a decline in insect and bird populations (Bouam et al. 2017, Bouam 2018). Agricultural land use has a significant impact on ecosystem services, as demonstrated in potato fields where it alters ecological interactions (Werling and Gratton 2010). While monocultures may offer short-term economic benefits, they often lead to reductions in biodiversity and disrupt ecosystem dynamics (Scarpa and Cicia 2000). Additionally, research conducted on La Réunion has shown that the intensity of agricultural practices influences predator species richness within mango agroecosystems (Jacquot et al. 2013).

Environmental degradation in the Mediterranean Region is exacerbated by the intensification of agricultural activities (Caraveli 2000). It is essential to acknowledge the adverse impacts of intensive agriculture to facilitate a shift towards agro-ecological systems (Duflot et al. 2022). Innovation is vital for assisting smallholder farmers in Africa to enhance production, while minimising environmental impacts (Juma et al. 2013). Agroecology promotes autonomy in resource use and emphasises functional diversity within production systems, aiming to enhance sustainability and ecosystem resilience (Baccar et al. 2018). It is crucial to prioritise production growth through productivity enhancement rather than by increasing inputs, while balancing fruit production with the conservation of natural resources (Demestihas et al. 2017). Therefore, to quantify management intensity and better understand its ecological impacts, the Agricultural Intensification (AI) Index provides a widely used framework that integrates factors, such as fertiliser and pesticide use, irrigation, mechanisation and crop diversity (Ruiz-Martinez et al. 2015). Globally, the AI Index has been applied to link agricultural practices with biodiversity, functional diversity and ecosystem services, allowing comparisons across different agroecosystems (Gebhardt et al. 2023).

In the Mediterranean Basin, AI has been common since the 1950s (Kizos and Koulouri 2006) and the area of land devoted to agricultural use has increased remarkably over recent centuries (Blondel and Aronson 1999). Perennial cropping systems have received comparatively less research attention than annual systems, despite notable differences in habitat structure and agricultural practices. Perennial crops typically exhibit more complex vegetation structure and are subject to lower levels of disturbance, which can have significant implications for biodiversity and ecological functioning (Matson et al. 1997, Bruggisser et al. 2010). AI impacts on biodiversity in North Africa are poorly studied. To our knowledge, aside from Bouam (2018), who examined olive groves, no research has focused on palm grove ecosystems, despite their unique location in harsh Saharan environments. This highlights a significant gap in ecological assessment in these agroecosystems. In this context, we hypothesise that moderate agricultural intensification within date palm groves promotes higher insect diversity by maintaining a balance between cultivated and semi-natural habitats.

In Algeria, to boost date production, farmers are increasingly adopting monovarietal cultivation practices, particularly focusing on the Deglet Nour variety. However, this trend may have significant consequences on biodiversity within these agroecosystems, particularly as the effects of AI on insect diversity remain largely underexplored. This study aims to assess the impact of AI on insect communities across varying intensification levels. Field surveys were carried out in three palm groves located in the Saharan region of Algeria (Ziban, Biskra). The research aims to determine whether agricultural intensification affects the diversity and abundance of insect groups within palm grove ecosystems. This would highlight the need to consider the effects of agricultural practices on local biodiversity and the challenges of maintaining sustainable agroecosystems linked to date palms while preserving biodiversity.

## Material and Methods

**Study area**

**Figure 1.**
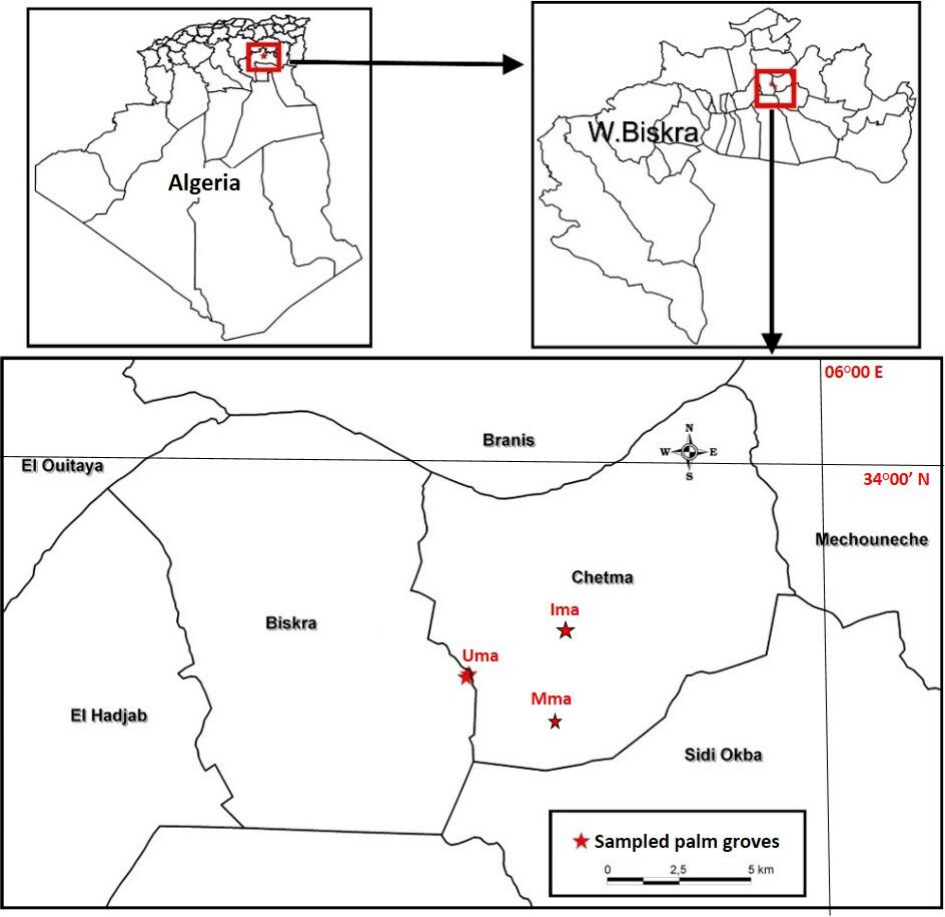
Geographical location of the three palm groves studied in the region of Biskra (north-east Algeria). (Uma: Unmanaged palm grove; Mma: Moderately managed; Ima: Intensively managed).

The investigation was carried out in three palm orchards located in Biskra Province (Ziban Region, north-east Algeria), in the Chetma area, which is known as the main centre for the production of ‘Deglet Nour’ dates in the country (Fig. 1). To minimise the influence of large-scale environmental heterogeneity, all orchards were selected within the same locality, where topographic and climatic variability is minimal. The three sites are situated 1.7 to 2.8 km apart, covering a compact area of approximately 24 km², which ensures comparable environmental conditions across orchards and limits the confounding effects of spatial variabilities. Meteorological data collected over the past 30 years showed a climate characterised by low and irregular precipitation, with an annual average of 107.57 ± 14.54 mm. April emerged as the rainiest month (24.6 ± 8.22 mm), while January recorded the lowest precipitation (1.0 ± 0.6 mm) and was also the coldest month (12.1 ± 0.1°C). The annual mean temperature was 23.1 ± 7.97°C, with July and August being the hottest months (34.9 ± 0.7°C and 34.7 ± 0.8°C, respectively). Relative humidity averaged 39.78 ± 9.38% and the mean wind speed was 11.11 ± 1.27 km/h.

**Evaluation of agricultural intensification**

To assess agricultural intensification in date palm cultivation, we calculated an Agricultural Intensification Index (IInAg), a tool widely used to compare production systems and evaluate their ecological impacts on biodiversity (Herzog et al. 2006, Flohre et al. 2011, Bouam 2018). We conducted a survey of 20 date producers in the region using a structured questionnaire covering key management practices, including pesticide application, mineral and organic fertilisation, irrigation methods, bagging, mechanical weeding and tillage. Based on the responses, eight farms were initially retained as potentially suitable for our study. Subsequent field visits allowed us to verify the accuracy of the reported practices, after which we selected three palm groves that fully met our criteria; all three cultivate the ‘Deglet Nour’ variety and represent distinct levels of agricultural intensification.

The IInAg used for assessing the AI for the three studied palm grove was calculated using the methods outlined by Herzog et al. (2006):

\begin{varwidth}{50in}\begin{equation*}
            IInAg = \frac{\sum_{i=1}^{n} \frac{(y_i - y_{\min})}{(y_{\max} - y_{\min})}}{n} \times 100
        \end{equation*}\end{varwidth}

The index is defined by observed values (y_i_), minimum (y_min_) and maximum (y_max_) values across orchards, with 'n’ representing the number of indicators. In our case, IInAg is calculated using seven indicators in the three sampled orchards: use of agrochemical products, use of fertilisers, use of organic manure, mechanical weeding interventions, plantation density, irrigation systems and the operation of bagging date stems. The main agrochemicals applied include Abamectin (18 g/l), used as an insecticide, acaricide and nematicide, as well as Cymoxanil (42 g/kg) and copper oxychloride (660 g/kg), both used as fungicides. All sampled orchards follow a monovarietal management system. (Table 1).

**Insect sampling and identification**

To sampling insect communities, we used differents traps: Barber pots, ground coloured traps and suspended coloured traps. For collecting insects by Barber pots, this consisted of using nine cylindrical pitfall traps (1 dm³), buried in a 400 m² plot, arranged in rows of three, 5 m apart. The Barber pots were filled with a mixture of water and detergent used as a preservative solution. Yellow traps (16 cm in diameter and height) were deployed, with four on the ground and four suspended from palm trees, all containing water and detergent. This sampling protocol was conducted monthly from September 2020 to August 2021. Trap contents were collected after 48 hours, preserved in 70% alcohol and identified at the laboratory LBBDD Biodiversity, Biotechnology and sustainable development (Batna 2 University) and the National Veterinary School in Algiers.

**Data analysis**

To illustrate the density of different insect populations, we calculated the total number of specimens collected in each type of groves (N). The diversity of insect communities in the three study palm groves, was evaluated by: the total taxa richness (S), which correspond to the total number of taxa recorded; and the Shannon diversity index H’ = -Ʃ Pi log_2_ Pi (where Pi = ni/N, the proportion by number of the taxa i) (Magurran 2004).

The diversity in three palm groves, in relation to AI, was assessed through total species richness (S) rarefaction-extrapolation curves, Shannon diversity indices and sampling coverage. Non-metric Multidimensional Scaling (NMDS) and Generalised Linear Mixed Models (GLMM) were performed in order to analyse the effect of AI on insect communities. All statistical analyses were performed using R software Core Team 2015.

## Results

**Diversity and abundance of insect communities according to the level of agricultural intensification**

This study allowed identifying 5,633 insect specimens, representing 267 species, 195 genera, 93 families and 11 orders. Moderately managed palm grove (MMa) showed the highest values across all biodiversity metrics, with a total abundance of 2,624 individuals, species richness (S) of 184 and a Shannon diversity index (H') of 5.42. In comparison, the intensively managed palm grove (IMa) and the unmanaged palm grove (UMa) ranked second and third, respectively for the three parameters dominated by the moderately intensified palm grove (Table 2).

**Figure 2.**
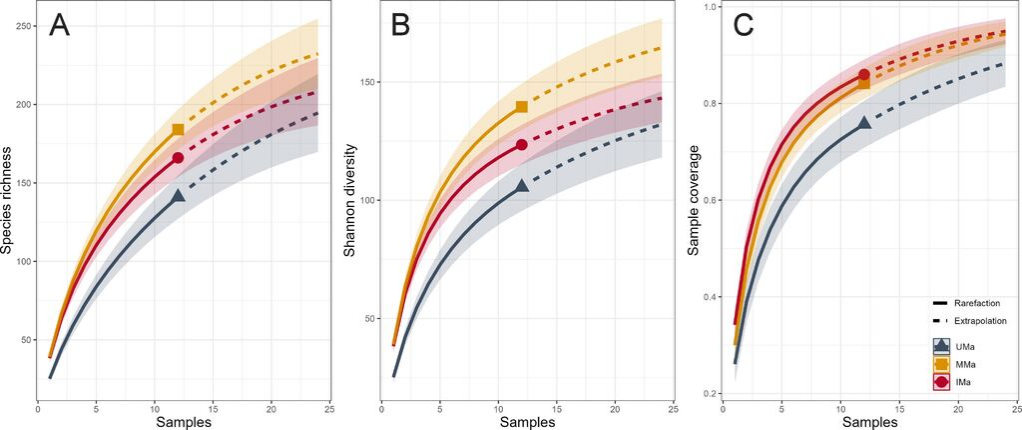
Rarefaction curves of observed species richness as a function of the number of surveys conducted. **A** Shannon diversity index; **B** Sample coverage rate; **C** At three levels of agricultural intensification (Unmanaged: UMa; Moderately managed: MMa; Intensively managed: IMa).

The confidence intervals for MMa and IMa show a degree of overlap, suggesting a relative similarity in species richness (Fig. 2A) and Shanon index diversity (Fig. 2B). The sampling coverage rate indicates a satisfactory rate (over 75 percent) for all three palm groves (Fig. 2C).

**Figure 3.**
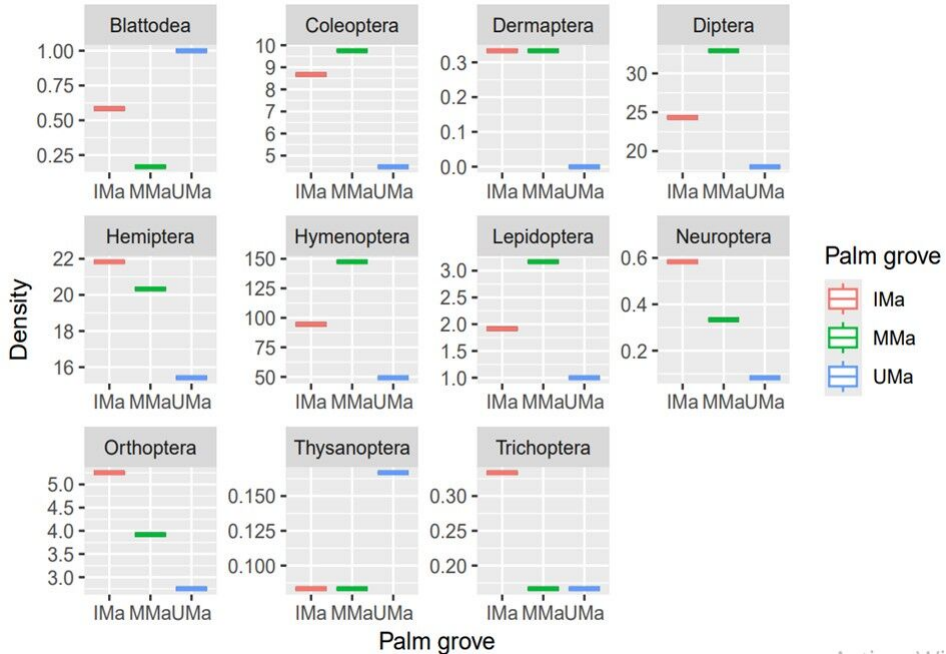
Density of insect orders according to agricultural intensification level in three palm groves (Unmanaged: UMa; Moderately managed: MMa; Intensively managed: IMa).

The abundance of the different orders of insects recorded varies from one type of palm grove to another depending on its degree of AI. Coleoptera, Hymenoptera, Lepidoptera and Diptera exhibited the highest density in the moderately managed palm grove (MMa). Orthoptera, Hemiptera, Neuroptera and Trichoptera were the most abundant in intensively managed orchard (IMa). Thysanoptera and Blattoptera were dominant in unmanaged orchards (UMa). Dermaptera is the order whose numbers were roughly the same in MMa and IMa (Fig. 3).

**Figure 4.**
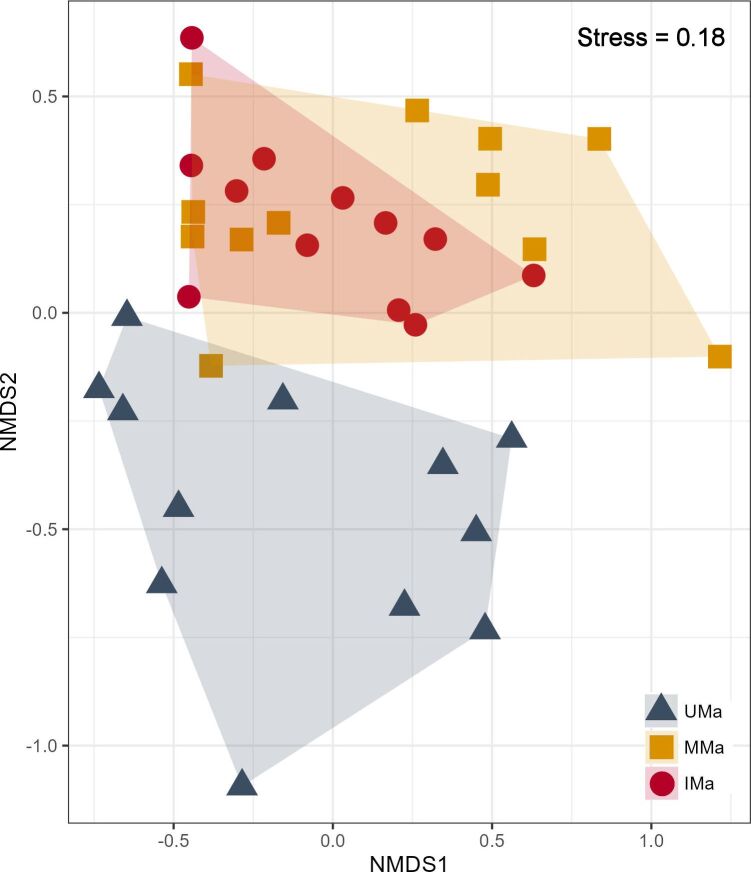
Ordination diagrams of the results from the non-metric multidimensional scaling (NMDS) analysis of three palm groves according to the level of agricultural intensification. (Unmanaged: UMa; Moderately managed: MMa; Intensively managed: IMa).

The NMDS analysis reveals a clear distinction between the insect community in UMa palm grove and that of MMa and IMa palm groves. Meanwhile, MMa and IMa insect communities exhibit significant overlap, indicating similar compositions (Fig. 4).

**Impact of agricultural intensification on species richness and abundance of insect communities**

**Figure 5.**
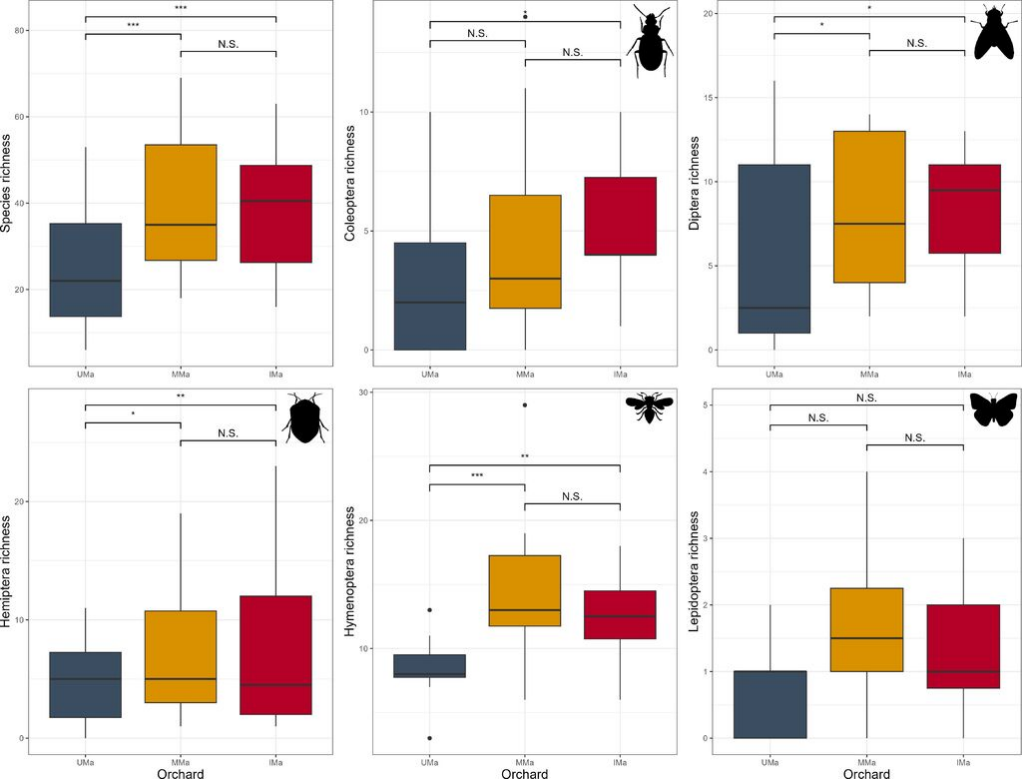
Results of the GLMM testing the correlations of species richness for all species combined (top left), as well as for the main orders of insects recorded in the palm groves studied. p-values: *p < 0.05; **p < 0.01; ***p < 0.001.

The GLMM analysis applied for five of the 11 orders of insects listed (Coleoptera, Diptera, Hemiptera, Hymenoptera and Lepidoptera) indicated significant differences in total species richness for the entire community of insects recorded and for all the insect orders amongst the UMa, MMa and IMa palm groves. No significant difference was noted between MMa and IMa whether for all insects or for the five insect orders considered in this analysis. For Coleoptera, significant differences were found between UMa and IMa, but not between UMa and MMa or MMa and IMa. Diptera exhibited significant differences amongst the three palm groves. Hemiptera and Hymenoptera showed significant differences between UMa, MMa and IMa, with no difference between MMa and IMa (Fig. 5).

**Figure 6.**
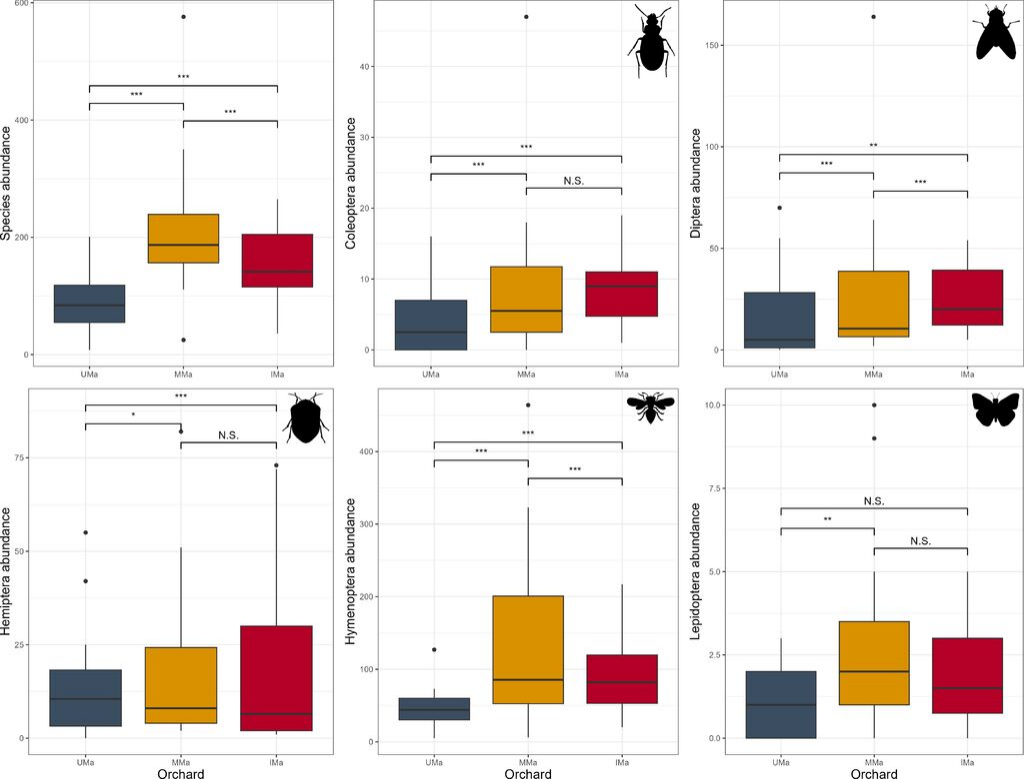
Results of the GLMM testing the correlations of abundance for all species combined (top left), as well as for the main orders of insects recorded in the palm groves studied. p-values: *p < 0.05; **p < 0.01; ***p < 0.001.

The GLMM analysis applyed for the variation of abundance of the total insects revealed highly significant differences amongst the UMa palm grove, MMa grove and IMa grove, including between MMa and IMa. For Coleoptera, significant differences were noted amongst the three palm groves, with no difference between MMa and IMa. Diptera exhibited highly significant differences across all palm groves. Hemiptera showed significant differences between UMa and MMa and between UMa and IMa, but not between MMa and IMa. Hymenoptera also displayed highly significant differences in all palm groves. For Lepidoptera, a significant difference was found between UMa and MMa, with no difference between UMa and IMa or between MMa and IMa (Fig. 6).

## Discussion

**Diversity and abundance of insects according to agricultural intensification**

This study shows that AI in Saharan climate appears to favour the abundance and diversity of insects. Despite the scope of this study being limited to a single year and a few sites, it provides valuable insights into how moderate agricultural intensification promotes insect diversity in Saharan palm groves. Our results are largely consistent with studies conducted in other arid and semi-arid regions, where moderate agricultural intensification has been shown to enhance insect diversity by increasing habitat heterogeneity and resource availability (e.g. Zergoun et al. 2020, El Harche et al. 2023).

However, many studies around the world have shown the opposite, highlighting that insect diversity has significantly decreased since the 1950s, primarily due to intensive agriculture and pesticide use (Raven and Wagner 2021) and the decline of insects in Europe is exacerbated by intensive agriculture and climate change (Habel et al. 2019). The low diversity of insects in the non-intensified palm grove is because biodiversity is generally low in arid and Saharan environments, but they present a composition of original species, adapted to these sometimes-extreme conditions. Furthermore, moderately and highly intensified palm groves provide new habitats and new ecological niches allowing the installation and acclimatisation of a great number of species, but of a generalist and opportunistic nature. Butler et al. (2012) highlight that, while the application of synthetic fertilisers may enhance insect populations, their effects are highly context-dependent and can potentially intensify interspecific competition within communities.

The moderately managed palm grove revealed that moderate intensification could enhance biodiversity. Active management promotes regeneration and introduction of new species, aligning with sustainable agricultural practices. Appropriate fertilisation and maintenance, along with careful pesticide use, create an environment conducive to insect biodiversity. This resource enrichment is a key mechanism underlying the observed increase in insect diversity under moderate intensification providing greater variety of food and habitats. In addition, intermediate disturbance contributes to higher diversity in the MMa. Low-to-moderate levels of disturbance prevent competitive exclusion by dominant species, while avoiding the habitat degradation typical of high-intensity management. In contrast, the intensively managed palm grove showed higher species richness than the UMa, but lower diversity than the MMa, indicating that excessive intensification can harm biodiversity by reducing habitats for other species. The high use of agrochemicals in the IMa, compared to the MMa, exacerbates this decline.

Non-metric multidimensional scaling (NMDS) analysis revealed the substantial impact of AI on insect biodiversity. The UMa exhibits reduced insect diversity, likely due to limited natural habitat, while the MMa retains richer habitat characteristics despite some intensification. Although the IMa shares similar insect composition with the MMa, it shows signs of potential species loss related to pesticide and chemical fertiliser use. Intensive agriculture and pesticide application lead to a decline in arthropod populations, threatening their ecological role (Vasconcelos 2022).

Certain orders, such as Hymenoptera, Diptera, Lepidoptera and Coleoptera, seem to benefit from moderate intensification. This may be explained by the fact that these most widespread and numerous groups of insects can take advantage of the available food resources in the moderately managed palm grove, while maintaining favourable habitats. In fact, moderate AI (e.g. organic amendments, diversified cropping, and reduced pesticide use) can increase plant productivity and floral resources, which, in turn, may support greater insect abundance and diversity, especially pollinators and natural enemies (Sutter et al. 2017). For example, Hymenoptera, which includes bees, can benefit from floral diversity in moderately intensive date palm agroecosystems. Hemiptera and Neuroptera, which respond positively to increased intensification, underscore the importance of the ecological specificities of each insect order. Orthoptera, Hemiptera and Neuroptera seem to adapt to the highly intensified orchard and manage to develop well in the conditions of such habitat. According to Löffler and Fartmann (2017), intensive grassland exploitation and reforestation harm orthopterans. The abundance of Thysanoptera in unmanaged palm grove indicates the sensitivity of this order and its preference for a natural, little-anthropogenised habitat. The abundance of Trichoptera in IMa cannot give a precise idea of their preferred environment given that this order is represented by species whose larval development takes place in an aquatic environment and their presence in IMa is mainly due to the proximity of a watercourse to the highly intensified palm grove.

**Impact of agricultural intensification on diversity and abundance of insects**

Our analysis indicates that AI significantly influences insect species richness. Low AI, such as that found in the UMa palm grove, supports diverse insect groups, while the MMa palm grove provides a more suitable habitat than the IMa palm grove. Coleoptera, Diptera, Hemiptera and Hymenoptera are considerably affected by higher AI, whereas, Lepidoptera exhibit resilience to variations in intensification. This goes against the results of several works indicating that pesticides and herbicides have a negative impact on biodiversity and pollinators (Strandberg et al. 2019, Overton et al. 2021). Diptera, which are essential for pollination, are experiencing a global decline due to environmental changes and high agricultural intensity is correlated with a decrease in Diptera diversity (Powell et al. 2024). AI reduces pollinator diversity and their pollination services (Deguines et al. 2014). The excessive use of agrochemicals threatens insects, while organic farming could significantly enhance biodiversity (Marliac et al. 2015).

The study highlights the importance of balanced management of palm groves, where moderate intensification can promote biodiversity, while excessive or absent intensification may harm insect abundance, particularly for Diptera and Hymenoptera. These findings are consistent with those of Attwood et al. (2008), which demonstrate a greater abundance of arthropods in low-intensity areas. Insects are generally more abundant in forest reserves than in agricultural zones (Weisner 2018).

Furthermore, AI in palm groves generally promotes the abundance of insects, but this impact differs from one order of insects to another. Barrett et al. (2023) showed that climate change and AI contribute to the decline in the abundance of pollinating insects. Intensive agricultural practices reduce the abundance and diversity of local wild species (Teyssedre 2022) and affects the abundance of beetles under both ecological and conventional farming conditions (Langraf et al. 2023).

In our study, the analysis of insect abundance reveals interesting trends related to their ecological adaptations in response to environmental changes. Coleoptera, Lepidoptera and Diptera benefit from moderate intensification due to their ability to adapt to various ecological niches. Hemiptera also show good adaptation, owing to their lifestyle, based on the consumption of sap and organic matter. According to Zhao et al. (2015), the increasing application of nitrogen fertilisers has led to higher population densities of cereal aphids. In contrast, Dermaptera, Neuroptera and Trichoptera exhibit low densities in intensified palm groves, indicating increased sensitivity to changes in intensification, likely due to their specific habitat requirements. Hymenopterans, although adapted to moderately intensive environments, experience a decline in abundance with increased intensification, which could adversely affect their reproduction. Orthoptera benefit from modified habitat structure, while Blattodea show variable densities, reflecting some plasticity in response to environmental changes, but also a dependence on specific conditions. According to Emmerson et al. (2016), AI leads to a decrease in the density of many organisms associated with agricultural landscapes.

Despite the fact that the work was carried out on a limited number of sites and over a period of one year, it has highlighted the impact of AI on entomological biodiversity in Saharan agrosystems. However, it would be useful to launch similar studies covering larger areas and spanning several years.

## Conclusions

This study provides new insights into how agricultural intensification shapes insect diversity and community structure in Saharan date palm groves. Our findings demonstrate that moderate intensification supports the highest levels of diversity, with a Shannon index exceeding 5.4 and significantly enhances insect abundance compared to both highly intensified and unmanaged groves. This pattern was particularly pronounced amongst key ecological groups, such as Coleoptera, Diptera and Hymenoptera, highlighting their sensitivity to management practices and their role as bioindicators in arid agroecosystems. Although the majority of surveyed farmers reported using organic fertilisers, the growing reliance on chemical pesticides poses emerging ecological risks, emphasising the need for precautionary approaches. The results collectively underline the importance of balanced, environmentally responsive management, which maintains habitat heterogeneity and supports beneficial insect communities.

## Figures and Tables

**Table 1.** Coordinates, values of the indicators and the agricultural intensification index calculated in the three studied palm groves.

**Characteristics and operations**	**Unmanaged palm grove UMa**	**Moderately managed Palm grove MMa**	**Intensively managed palm grove IMa**
Latitude	34°50'54.57"N	34°50'6.10"N	34°50'56.34"N
Longitude	5°46'0.41"E	5°47'29.81"E	5°47'50.55"E
Altitude (a.s.l. metres)	97	82	84
Surface (ha)	5	4	4
Number of uses of agrochemical products/year	0	2	4
Quantity of fertilisers used (kg/year)	0	147.63	278.00
Quantity of organic manure used (kg/year)	0	1628.00	8148.00
Number of mechanical weeding interventions/year	0	1	1
Plantation density (palms/hectare)	120	111	139
Irrigation system (presence/absence)	0	1	1
Operation of date stems bagging (presence/absence)	0	0	1
InAg index value	**4.59**%	**46.15**%	**100**%
Level of agricultural intensification	**Low**	**Medium**	**High**

**Table 2.** Abundance and diversity parameters of insect communities according to agricultural intensification in palm groves.

**Parameter**	Unmanaged palm grove **UMa**	Moderately managed palm grove **MMa**	Intensively managed palm grove **IMa**
Abundance	1108	2624	1901
Taxa richness S	141	184	167
Shannon index H'	4.93	5.42	5.30
